# The Definition of Lymphocyte Activating Factor: Giving a Helping Hand to Serendipity

**DOI:** 10.3389/fimmu.2014.00610

**Published:** 2014-11-28

**Authors:** Igal Gery

**Affiliations:** ^1^Laboratory of Immunology, National Eye Institute, National Institutes of Health, Bethesda, MD, USA

**Keywords:** interleukin-1, lymphocyte activating factor, cytokines, macrophages, thymocytes

The idea that soluble cell products play important roles in the complex process of the immune response was supported by scientific evidence in the sixties in several publications including those by David et al. (1) and Bloom and Bennett (2), who discovered the activity of macrophage migration inhibitory factor (MIF), Gordon and McLean (3) who reported that media of leukocyte cultures contain mitogenic factors, Kasakura and Lowenstein (4), who showed the activity of a “blastogenic factor” and Ruddle and Waksman (5), who discovered the activity of lymphotoxin. Further, a 1970 paper by Bach et al. (6) reported on a soluble factor that could replace macrophages in purified lymphocyte proliferative responses.

Unlike the factors mentioned above, the definition of lymphocyte activating factor (LAF) was a result of a study aimed at an entirely different purpose. In May 1970, I came to the lab of Byron Waksman, at Yale University, for a sabbatical from my position at the Hebrew University Medical School. Prior to coming to Yale I acquired expertise in culturing lymphocytes and Richard (Dick) Gershon, who collaborated with Waksman’s group, approached me with the idea to use this expertise for studying the activity of the “suppressor cells” he discovered. It is of note that the concept of a population of lymphocytes whose function is suppression of immune responses by other lymphocyte populations was revolutionary at that time and Dick had to struggle to get his data published; his seminal paper on suppressor cells was finally accepted by *Immunology* (7). The experiment Dick suggested was to inject naïve mice with large (“tolerizing”) doses of sheep red blood cells (RBC) and to test lymphocytes from these treated mice for their proliferative responses in culture to the mitogen phytohemagglutinin (PHA) in the presence of sheep RBC. We focused on spleen lymphocytes of the treated mice, but we also examined the response of thymus cells of the injected mice. For specificity controls we used human whole blood cells (from a colleague donor). The mouse spleen cultures exhibited a moderate level of suppressed response to PHA (8). In addition, however, I noticed an unexpected response: vigorous proliferation by thymus cell cultures incubated with human RBC (our control…) and PHA. The thymocytes did not respond, however, to PHA alone. Further analysis of the unexpected finding established that this was not a fluke and revealed that the thymocyte response was actually triggered by the small number of leukocytes that “contaminated” the fresh human RBC preparation. Indeed, when using purified human blood leukocytes, as few as 6,000 of these cells, along with PHA, were sufficient to stimulate a significant response by the thymocytes. The next step was, obviously, to examine the supernatant of human leukocytes for stimulatory activity and I was delighted to find that the supernatant had strong stimulatory activity on the thymocyte response to PHA. We believed that these preliminary observations were of sufficient importance and summarized them as a short communication in the *Journal of Immunology* (9).

During the following months of my sabbatical at Yale I expanded the research, focusing on two related issues: the analysis of the responding cells and of the cells that produce the stimulating factor. At that stage we had to give the factor a name and we chose “Lymphocyte activating factor” or “LAF”. The best responding cells were identified to be the more mature thymocytes, but LAF also stimulated the response of less mature thymocytes, as well as the response to stimulants by murine spleen cells. Useful information was particularly provided by the analysis of the LAF producing cells and their stimulants. High levels of LAF activity were secreted by adherent cells (mostly macrophages) stimulated by lipopolysaccharide (LPS) and by non-adherent lymphocytes stimulated with PHA or concanavalin A. In hindsight, it seems that the macrophages produced LAF activity, whereas the PHA-stimulated lymphocytes released mainly another stimulatory factor. It is also noteworthy that the LPS used in these preliminary experiments was a gift from the lab of Elisha Atkins, two floors below our lab. Elisha and his group had been using LPS as a stimulant for the production of the endogenous pyrogenic factor they measured by inducing fever in animals. That batch of LPS was also used in a study we carried out at the same time showing for the first time that LPS is mitogenic for B-cells (10).

Before leaving New Haven I left with Byron Waksman drafts for two manuscripts that summarized the data. Byron rewrote the manuscripts that were accepted for publication by the *Journal of Experimental Medicine*. The first paper (11) is co-authored by Byron Waksman and Dick Gershon, but Dick was not included in the authors’ list of the second one (12). This was clearly unfair to Dick and I certainly feel badly about it.

During my last months in New Haven I also contacted Bob Handschumacher, at the Department of Pharmacology at Yale, to help me with the characterization of LAF. We carried out some preliminary experiments, but in order to complete the study I returned to New Haven in the summer of 1972 and together with Bob we collected some basic information on the factor, including the finding that LAF is a protein, with a size of ~15 kDa. We submitted a manuscript that summarized the data to the *Journal of Immunology*, but the manuscript was rejected. We then submitted it to *Cellular Immunology* (13) and were delighted to learn that this paper was subsequently highly cited.

Later studies by other groups [e.g., Ref. (14)] revealed that highly purified “LAF” preparations functioned to promote the production of a T cell-derived activity, which was the basis for the interleukin nomenclature, first proposed at the Second International Lymphokine Workshop in 1979 at Ermatigen Switzerland. Thus, “interleukin-1 (IL-1)” made by macrophages was distinguishable from “IL-2”, made by lymphocytes (14).

Looking back, I feel lucky for including the human “RBC” control cultures in the initial experiment performed 44 years ago and proud for pursuing the unexpected and weird result by the subsequent experiments that yielded the definition of the LAF.

## Conflict of Interest Statement

The author declares that the research was conducted in the absence of any commercial or financial relationships that could be construed as a potential conflict of interest.

## References

[1] David JR Al-Askari S Lawrence HS Thomas L Delayed hypersensitivity in vitro. I. The specificity of inhibition of cell migration by antigens. J Immunol (1964) 93:264–73. 14205621

[2] Bloom BR Bennett B. Mechanism of a reaction in vitro associated with delayed-type hypersensitivity. Science (1966) 153(3731):80–2. 10.1126/science.153.3731.80 5938421

[3] Gordon J MacLean LD A lymphocyte-stimulating factor produced in vitro. Nature (1965) 208(5012):795–6 10.1038/208795a0 4223737

[4] Kasakura S Lowenstein L A factor stimulating DNA synthesis derived from the medium of leukocyte cultures. Nature (1965) 208(5012):794–5 10.1038/208794a0 5868897

[5] Ruddle NH Waksman BH. Cytotoxicity mediated by soluble antigen and lymphocytes in delayed hypersensitivity. 3. Analysis of mechanism. J Exp Med (1968) 128(6):1267–79. 10.1084/jem.128.6.1237 5693925 PMC2138574

[6] Bach FH Alter BJ Solliday S Zoschka DC Janis M Lymphocyte reactivity in vitro. II Soluble reconstituting factor permitting response of purified lymphocytes. Cell Immunol (1970) 1(2):219–27 10.1016/0008-8749(70)90009-2 5523579

[7] Gershon RK Kondo K Infectious immunological tolerance. Immunology (1971) 21(6):903–14. 4943147 PMC1408252

[8] Gershon RK Gery I Waksman BH Suppressive effects of in vivo immunization on PHA responses in vitro. J Immunol (1974) 112(1):215–21. 4130148

[9] Gery I Gershon RK Waksman BH Potentiation of cultured mouse thymocyte responses by factors released by peripheral leucocytes. J Immunol (1971) 107(6):1778–80. 4941117

[10] Gery I Kruger J Spiesel SZ Stimulation of B-lymphocytes by endotoxin. Reactions of thymus-deprived mice and karyotypic analysis of dividing cells in mice bearing T 6 T 6 thymus grafts. J Immunol (1972) 108(4):1088–91. 5023169

[11] Gery I Gershon RK Waksman BH. Potentiation of the T-lymphocyte response to mitogens. I. The responding cell. J Exp Med (1972) 136(1):128–42. 10.1084/jem.136.1.128 5033417 PMC2139184

[12] Gery I Waksman BH. Potentiation of the T-lymphocyte response to mitogens. II. The cellular source of potentiating mediator(s). J Exp Med (1972) 136(1):143–55. 10.1084/jem.136.1.143 5033418 PMC2139186

[13] Gery I Handschumacher RE Potentiation of the T lymphocyte response to mitogens. III. Properties of the mediator(s) from adherent cells. Cell Immunol (1974) 11(1–3):162–9 10.1016/0008-8749(74)90016-1 4549027

[14] Smith KA Lachman LB Oppenheim JJ Favata MF. The functional relationship of the interleukins. J Exp Med (1980) 151(6):1551–6. 10.1084/jem.151.6.1551 6770028 PMC2185867

